# Neural Entrainment to Auditory Imagery of Rhythms

**DOI:** 10.3389/fnhum.2017.00493

**Published:** 2017-10-13

**Authors:** Haruki Okawa, Kaori Suefusa, Toshihisa Tanaka

**Affiliations:** ^1^Department of Electrical and Electronic Engineering, Tokyo University of Agriculture and Technology, Tokyo, Japan; ^2^Department of Electrical and Information Engineering, Tokyo University of Agriculture and Technology, Tokyo, Japan; ^3^RIKEN Brain Science Institute, Saitama, Japan

**Keywords:** rhythm perception, EEG analysis, discrete Fourier transform, machine learning, canonical correlation analysis

## Abstract

A method of reconstructing perceived or imagined music by analyzing brain activity has not yet been established. As a first step toward developing such a method, we aimed to reconstruct the imagery of rhythm, which is one element of music. It has been reported that a periodic electroencephalogram (EEG) response is elicited while a human imagines a binary or ternary meter on a musical beat. However, it is not clear whether or not brain activity synchronizes with fully imagined beat and meter without auditory stimuli. To investigate neural entrainment to imagined rhythm during auditory imagery of beat and meter, we recorded EEG while nine participants (eight males and one female) imagined three types of rhythm without auditory stimuli but with visual timing, and then we analyzed the amplitude spectra of the EEG. We also recorded EEG while the participants only gazed at the visual timing as a control condition to confirm the visual effect. Furthermore, we derived features of the EEG using canonical correlation analysis (CCA) and conducted an experiment to individually classify the three types of imagined rhythm from the EEG. The results showed that classification accuracies exceeded the chance level in all participants. These results suggest that auditory imagery of meter elicits a periodic EEG response that changes at the imagined beat and meter frequency even in the fully imagined conditions. This study represents the first step toward the realization of a method for reconstructing the imagined music from brain activity.

## 1. Introduction

Auditory perception is one of the fundamental functions of human brain. Several studies have investigated the reconstruction of auditory images by measuring brain activities while a human is perceiving auditory stimuli or imaging speech. In the speech domain, silent speech interfaces, which allow speech communication without vocalization, have been studied intensively in recent years (Denby et al., 2010; Matsumoto and Hori, 2014; Yamaguchi et al., 2015; Yoshimura et al., 2016). In terms of music perception, Schaefer et al. (2011) reported that it was possible to detect which one out of seven types of musical styles the participant was listening to based on brain activity. However, a method for reading the specific structure of perceived music from brain activity has not been established. Thus, as a first step toward the reconstruction of tangible music, it seems natural to investigate the reconstruction of perceived rhythm.

When listening to music, a human can naturally perceive rhythm. Sensorimotor synchronization, such as tapping, dancing, and marching, is the rhythmic coordination of perception and movement (Repp, 2005; Repp and Su, 2013). Rhythm is one of the main elements of music, and its structure is determined only by a cycle period in the simplest case. Hence, it is relatively easy to decode rhythm compared to the other elements of music, such as melody and harmony. Typically rhythm often refers to one of its sub-constituent elements such as beat and meter (Thaut et al., 2014). In the literature, beat is often used as a term which refers to one of a series of perceived pulses which are subjectively equal units in the temporal sequences (Large and Kolen, 1994; Kotz et al., 2009), and meter is traditionally defined as the number of beats between more or less regularly accented tones (Cooper and Meyer, 1960).

The neural mechanisms of rhythm processing have been studied for many years. The resonance theory (Large and Kolen, 1994; van Noorden and Moelants, 1999; Large, 2008; Large and Snyder, 2009) explains that beat and meter perception arise from neural oscillation resonating to rhythmic stimuli. This theory has been also supported by several studies that measure brain activity related to beat and meter perception (Large et al., 2015). Previous studies related to beat and meter perception have explored the event-related potentials (ERP) response to auditory rhythmic stimuli. The ERP, which is a response to an external stimulus, enables researchers to capture brain activity related to sensory, cognitive, or motor events (Pritchard, 1981; Demiralp et al., 1998; Donchin et al., 2000; Sur and Sinha, 2009). The ERP responses to rhythmic violations vary between strong and weak beats (Brochard et al., 2003; Snyder and Large, 2005; Potter et al., 2009), and results of previous studies suggest that brain activity reflects the perceived metric structure. Schaefer et al. (2011) reported that when participants imagined accent patterns to periodic sounds, the ERP response to the accented sounds was different from that to the unaccented sounds. Nozaradan et al. (2011, 2012) investigated brain activity during rhythm perception using a different method than an ERP approach. They analyzed an amplitude spectrum of an electroencephalogram (EEG) and reported that neural entrainment to beat and meter can be captured as a steady-state evoked potential (SSEP), which is a response to a periodic stimulus (Galambos et al., 1981; Stapells et al., 1984; Plourde, 2006; Vialatte et al., 2009; Zhu et al., 2010). The researchers recorded EEG while participants listened to periodic sound and imagined the meters (binary and ternary) of perceived beats. The results revealed that meter imagery elicited a periodic EEG response tuned to the meter frequency (Nozaradan et al., 2011, 2012). Moreover, although the rhythmic stimuli can elicit the ERP, the stimuli can contaminate the EEG response to the meter imagery. In other words, there is a possibility that the brain response to the stimulus and to the meter imagination may not be separated. Therefore, it is crucial to investigate neural entrainment during meter imagery without any auditory stimulus. Although in the absence of any auditory stimulus, Jomori et al. (2011) observed relative negative potentials in the EEG during beat imagery (not meter imagery), they did not focus on neural entrainment related to the SSEP suggested by Nozaradan et al. (2011, 2012).

**Figure 1:**
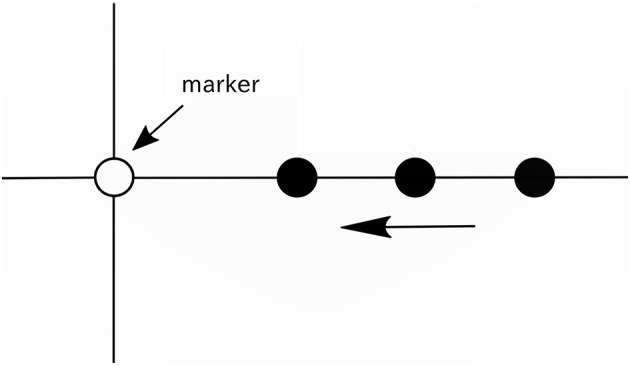
Illustration of the movie. A vertical line, horizontal line, white circle (marker), and black circle were displayed on a screen. The black circles appeared from the right end of the screen at intervals of 416 ms (approximately 2.4 Hz), and each black circle moved to the left on the horizontal line at a constant speed and disappeared when it reached the left end of the screen.

We hypothesized that without the auditory stimulus, neural entrainment to the imagined beat and meter could be captured in the EEG. In this study, we recorded EEG while participants imagined three types of rhythm (unaccented beat, binary meter, and ternary meter) synchronized with visual timing. We asked participants to imagine tones at regular intervals for beats imagery. For the meter imagery, we asked participants to imagine a metric structure in which specific beats were mentally accented. For synchronization of beat imagery with EEG recordings, a movie (see Figure 1 and Supplementary Video 1), or rhythm game, is shown to participants instead of auditory stimuli (Nozaradan et al., 2011, 2012). We also recorded EEG while the participants only gazed at the visual timing (movie) to confirm the visual effects of the movie. Furthermore, aiming a potential application to brain machine interfacing (BMI), we performed classification of imagined rhythm from a single trial EEG to verify that brain activities differ one type of rhythm imagery to another.

## 2. Methods

### 2.1. Participants

Nine participants (eight males and one female; mean age = 21.8 years, SD = 0.78) in their twenties participated in the experiment. All participants were healthy and had normal or corrected-to-normal vision. They provided written informed consent, and the study was approved by the research ethics committee of Tokyo University of Agriculture and Technology.

### 2.2. Movie

During recording, the movie (see Supplementary Video 1) was displayed on a screen to provide participants the reference tempo for beat imagery. As shown in Figure 1, a vertical line, horizontal line, white circle, and a series of moving black circles were displayed on a screen. In this paper, the white circle is called a “marker.” The black circles appeared from the right end of the screen at intervals of 416 ms (approximately 2.4 Hz), and each black circle was scrolled from the right to the left along the horizontal line at a constant speed and disappeared when it reached the left end of the screen. The black circles overlapped the marker one after another at intervals of 416 ms, and this time interval was the reference tempo for beat imagery. The duration of the movie was 12 s. In the movie, 25 black circles appeared in total. The movie was generated using the PsychToolbox running under MATLAB® (MathWorks).

### 2.3. Experimental design

**Figure 2:**
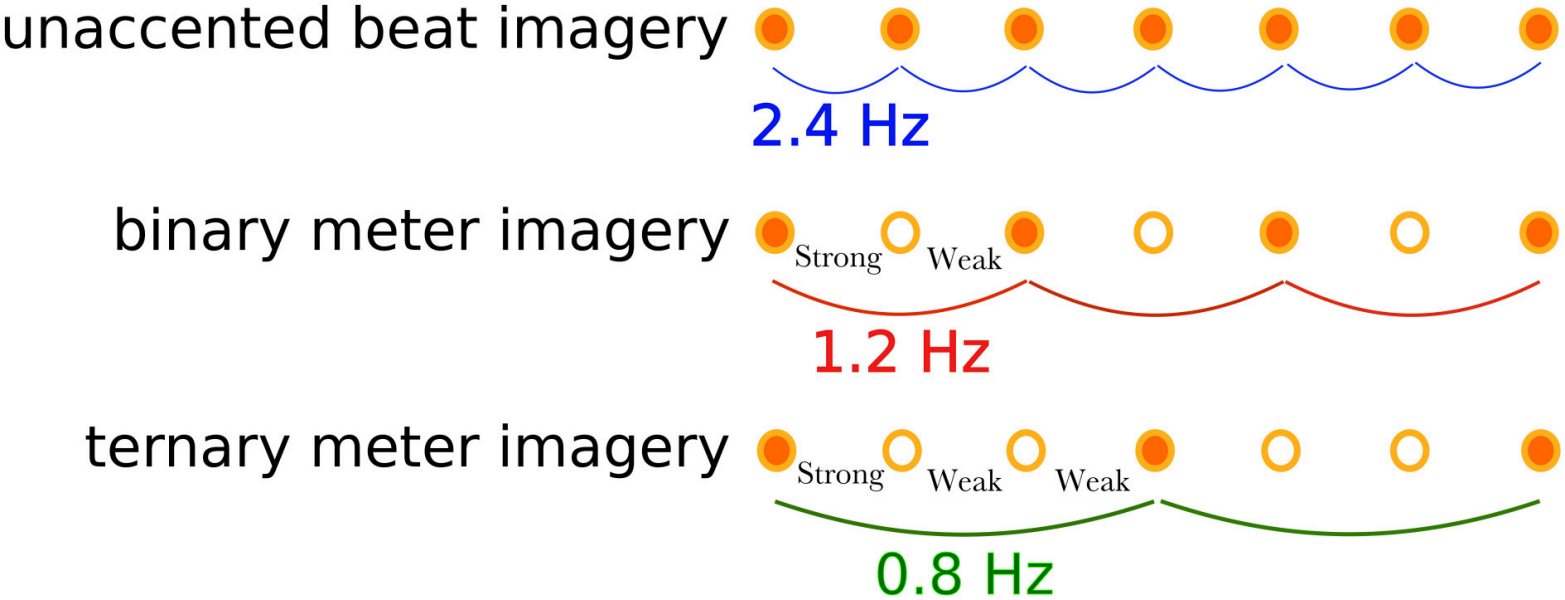
Illustration of the imagined rhythm structures for the three tasks. The unaccented beat imagery is the imagined isochronic beat of a frequency of 2.4 Hz without accents. The binary meter imagery consists of two accents: strong-weak, resulting in the meter frequency of 1.2 Hz. The ternary meter imagery has another accecent pattern: strong-weak-weak, resulting in the meter frequency of 0.8 Hz.

Participants were asked to perform a marker-viewing task and three types of rhythm imagery tasks: a unaccented beat imagery (defined as isochronous beat imagery without accenting) task, a binary meter (i.e., metrical accent patterns of strong-weak isochronous beats) imagery task, and a ternary meter (i.e., metrical accent patterns of strong-weak-weak isochronous beats) imagery task, as shown in Figure 2. Participants performed the tasks in the following order: marker-viewing, unaccented beat imagery, binary meter imagery, and ternary meter imagery. The order of the rhythmic imagery tasks were not randomized in order to match the experimental conditions of the previous work (Nozaradan et al., 2011), although there might have been potential of practice effects that could increase the performance on later tasks. There were several minutes of breaks between tasks to reduce fatigue. In the marker-viewing task, participants were asked to watch the movie without thinking about anything. This task was performed to confirm the visual effects of the movie and compare the results with the rhythm imagery tasks. Participants were instructed to declare if they were conscious of the beat, and we excluded the trial and executed it again in that case. In the rhythm imagery tasks, participants were asked to watch the movie and to imagine a tone at the moment each black circle overlapped the marker. Participants were presented with a pure tone of 333 Hz and memorized the pitch of the tone before the rhythm imagery tasks. They were then asked to imagine the tone during the rhythm imagery tasks for the beats imagery. In the unaccented beat imagery task, participants imagined beats without accenting as far as possible. In the binary meter imagery task, participants imagined a binary meter rhythm, which consisted of two beats, by mentally accenting every other imaged tone as “strong-weak-strong-weak-strong….” In the ternary meter imagery task, participants imagined a ternary rhythm, which consisted of three beats, by mentally accenting every third imaged tone as “strong-weak-weak-strong-weak-weak-strong….” Before the first trial of each meter imagery task, the instructed sequences of accented tones were presented to participants. The presented audio files are found in Supplementary Materials (Supplementary Audios 1, 2). To confirm the evidence of imagery, we adopted the following “error correction” scheme. In the binary and ternary meter imagery tasks, since participants were instructed to imagine the first tone as “strong,” they should imagine the last tone (the 25th tone) as “strong.” After a trial finished, they were instructed to declare whether or not they imagined the last tone as “weak,” which indicated that they failed to imagine the meter at some point, and thus we excluded the trial and executed it again.

Prior to each of the rhythm imagery tasks, participants practiced the aforementioned rhythm production by hand tapping. After the practice, the participants were tested if they could produce rhythm correctly. Each task consisted of 20 trials. For each trial, the movie was presented for 12 s. The EEG recording started at 2 s before the onset of the movie and stopped when the movie finished.

### 2.4. EEG recordings

**Figure 3:**
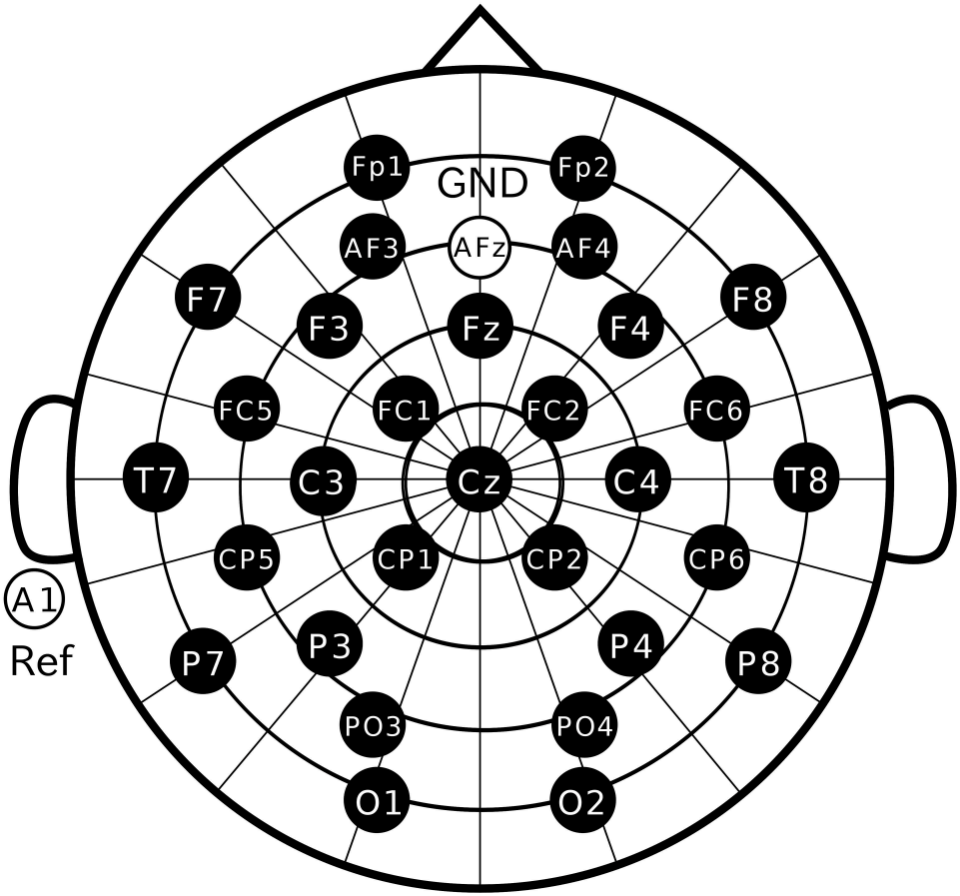
Electrode positions. Thirty electrodes were located at Fp1, Fp2, AF3, AF4, F3, F4, F7, F8, Fz, FC1, FC2, FC5, FC6, T7, T8, C3, C4, Cz, CP1, CP2, CP5, CP6, P3, P4, P7, P8, PO3, PO4, O1, and O2 following the international 10–10 system.

Participants were comfortably seated in a chair with their head resting on a support. They were instructed to keep their eyes fixated on the marker in the display and not allowed to move their body during recording. We used Ag/AgCl active electrodes, which were products of Guger Technologies (g.tec) named g.LADYbird, g.LADYbirdGND (for GND), and g.GAMMAearclip (for reference, earclip type), to record EEG data. These were driven by the power supply unit named g.GAMMAbox (g.tec). As illustrated in Figure 3, 30 electrodes were located at Fp1, Fp2, AF3, AF4, F3, F4, F7, F8, Fz, FC1, FC2, FC5, FC6, T7, T8, C3, C4, Cz, CP1, CP2, CP5, CP6, P3, P4, P7, P8, PO3, PO4, O1, and O2 following the international 10–10 system. The electrodes for GND and reference were placed on AFz and A1, respectively. The EEG signals were amplified by MEG-6116 (Nihon Kohden), which applied low-pass and highpass analog filters for each channel. The cutoff frequencies of the lowpass and the high-pass filters were set to 300 Hz and 0.08 Hz, respectively. The signals were sampled by an A/D converter (AIO-163202FX-USB, Contec) with a sampling rate of 3,000 Hz and recorded with the Data Acquisition Toolbox of the MATLAB® (MathWorks).

### 2.5. EEG signal preprocessing

EEG data were visually inspected to remove trials including artifacts. After the A/D conversion, EEG signals were re-referenced to the voltage averaged across all the electrodes. EEG signals were segmented into epochs lasting 10 s from the onset of beat imagery (at the time when the first black circle overlapped the marker). EEG epochs were filtered using a third-order Butterworth band-pass filter from 0.5 to 15 Hz to remove slow and fast drifts in the recorded signals. EEG epochs were segmented into four subepochs with a length of 2.5 s, which were then averaged.

### 2.6. EEG amplitude spectrum analysis

For each participant and task, the preprocessed EEG with a length of 2.5 s were averaged across trials. The obtained trial-average waveforms were transformed in the frequency domain using a discrete Fourier transform. The frequency resolution Δ*f* of the obtained amplitude spectrum ϕ(*f*) was 0.1 Hz. In order to remove background noise of the amplitude spectrum, at each frequency bin, we subtracted the average amplitude at neighboring frequency bins from the amplitude at given frequency *f*_*p*_:

(1) $$\begin{array}{r} \Phi \left(f_{p}\right)=\phi \left(f_{p}\right)-\frac{\sum_{n=1}^{2}\left\{\phi \left(f_{p}-n\Delta f\right)+\phi \left(f_{p}+n\Delta f\right)\right\}}{4} \end{array}$$

where Φ(*f*) is the noise-subtracted amplitude spectrum. The neighboring frequency bins were two frequency bins ranging from −0.2 to 0 Hz and +0.1 to +0.3 Hz relative to each frequency.

For each task, one-tailed, one-sample Wilcoxon signed-rank tests were conducted to determine whether EEG signal amplitudes at frequencies 0.8, 1.2, 1.6, and 2.4 Hz were significantly greater than zero. These statistical tests were applied to EEG spectral amplitudes averaged across all scalp electrodes. Frequencies of 2.4, 1.2, and 0.8 Hz correspond to the beat, the binary meter, and the ternary meter, respectively. It should be noted that the frequency of 1.6 Hz is the second harmonic of 0.8 Hz. The amplitude at the frequency of 1.6 Hz, which is the second harmonic of 0.8 Hz, was also analyzed, since Nozaradan et al. (2011) reported that the second harmonic was observed during the ternary meter imagery. In this paper, we refer to these four frequencies as target frequencies. Furthermore, to inspect the visual effects of the movie, one-tailed, one-sample Wilcoxon signed-rank tests were conducted to determine whether EEG spectral amplitude at the beat frequency (2.4 Hz) in each of the rhythm imagery tasks was significantly greater than that in the marker-viewing task. These statistical tests were applied to EEG spectral amplitudes averaged across the four electrodes in the occipital area (PO3, PO4, O1, O2) and the other 26 electrodes in the non-occipital area. The results were considered significant at a level of *p* < 0.10.

For each target frequency, a Friedman test was conducted to compare the effect of type of rhythm imagery on the EEG spectral amplitudes. The Friedman test was applied to EEG spectral amplitudes averaged across all scalp electrodes. When the Friedman test found a significant effect, the pairwise Wilcoxon signed-rank tests were also performed. The Holm method was used to counteract the problem of multiple comparisons. The results were considered significant at a level of *p* < 0.10.

### 2.7. Imagined rhythm classification based on machine learning technique

We performed machine learning-based classification of imagined rhythm from a single-trial EEG to verify that brain activities differ from one type of rhythm imagery to another. The purpose of this classification is to provide more support for the hypothesis that observed EEG signals under three types of rhythm imagery are distinct.

#### 2.7.1. Feature extraction

For feature extraction, we used canonical correlation analysis (CCA). CCA is the method for analyzing correlation between two multichannel signals (Hotelling, 1936). Considering two multichannel signals, CCA finds the linear combination coefficients that give the largest correlation between ***x***(*t*) and ***y***(*t*). Linear combinations of the two signals are denoted as $X\left(t\right)=w_{X}^{\text{T}}x\left(t\right)$ and $Y\left(t\right)=w_{Y}^{\text{T}}y\left(t\right)$, respectively, and CCA finds the weight vectors, ***w***_*X*_ and ***w***_*Y*_, which maximize the correlation between ***X***(*t*) and ***Y***(*t*) by solving the following problem:

(2) $$\begin{array}{rc} \underset{w_{X},w_{Y}}{\max}\rho & =\frac{E\left[X\left(t\right)Y\left(t\right)\right]}{\sqrt{E\left[X{\left(t\right)}^{2}\right]E\left[Y{\left(t\right)}^{2}\right]}}\\ =\frac{w_{X}^{\text{T}}E\left[x\left(t\right)y{\left(t\right)}^{\text{T}}\right]w_{Y}}{\sqrt{w_{X}^{\text{T}}E\left[x\left(t\right)x{\left(t\right)}^{\text{T}}\right]w_{X}w_{Y}^{\text{T}}E\left[y\left(t\right)y{\left(t\right)}^{\text{T}}\right]w_{Y}}}. \end{array}$$

The maximum of correlation coefficient ρ is called canonical correlation. This maximization problem can be solved by a generalized eigenvalue problem. We employed the feature extraction method for steady-state visually evoked potentials with CCA proposed by Lin et al. (2006), where signal ***x***(*t*) is the set of preprocessed EEG signals, and signal ***y***(*t*) is the set of reference signals that have the same length as ***x***(*t*). The reference signal ***y***_*f*_(*t*) is defined (Lin et al., 2006) as

(3) $$\begin{array}{r} y_{f}\left(t\right)={\left[\sin\left(2\pi ft\right),\;\cos\left(2\pi ft\right)\right]}^{\text{T}} \end{array}$$

and constructed by sine-cosine waves at the target frequency *f*. For *f* = 0.8, 1.2, 1.6, and 2.4 Hz, we calculated ρ_*f*_, which is defined as a canonical correlation between the multichannel EEG signal ***x***(*t*) and the reference signal ***y***_*f*_(*t*), and constructed a feature vector

(4) $$\begin{array}{r} z={\left[\rho_{0.8},\;\rho_{1.2},\;\rho_{1.6},\;\rho_{2.4}\right]}^{\text{T}}. \end{array}$$

The value of the canonical correlation ρ_*f*_ depends on frequency components contained in the EEG signal; for example, the canonical correlation ρ_0.8_ would be a larger value when the EEG signal includes a frequency component of 0.8 Hz. Such a feature extraction method has been used for frequency recognition of EEG (Lin et al., 2006; Kimura et al., 2013; Suefusa and Tanaka, 2017).

#### 2.7.2. Classification

We performed three-class classification (unaccented beat imagery vs. binary meter imagery vs. ternary meter imagery) and two-class classification (unaccented beat imagery vs. binary meter imagery, unaccented beat imagery vs. ternary meter imagery, and binary meter imagery vs. ternary meter imagery). Classification was performed using Support Vector Machine (SVM) (Boser et al., 1992), which is one of the most popular supervised machine learning techniques for classification. For three-class classification, the “one against one” approach was used to solve the multi-class problem (Knerr et al., 1990). To evaluate the classification accuracy, we applied leave-one-out cross-validation (Kohavi, 1995).

## 3. Results

### 3.1. EEG amplitude spectra

**Figure 4:**
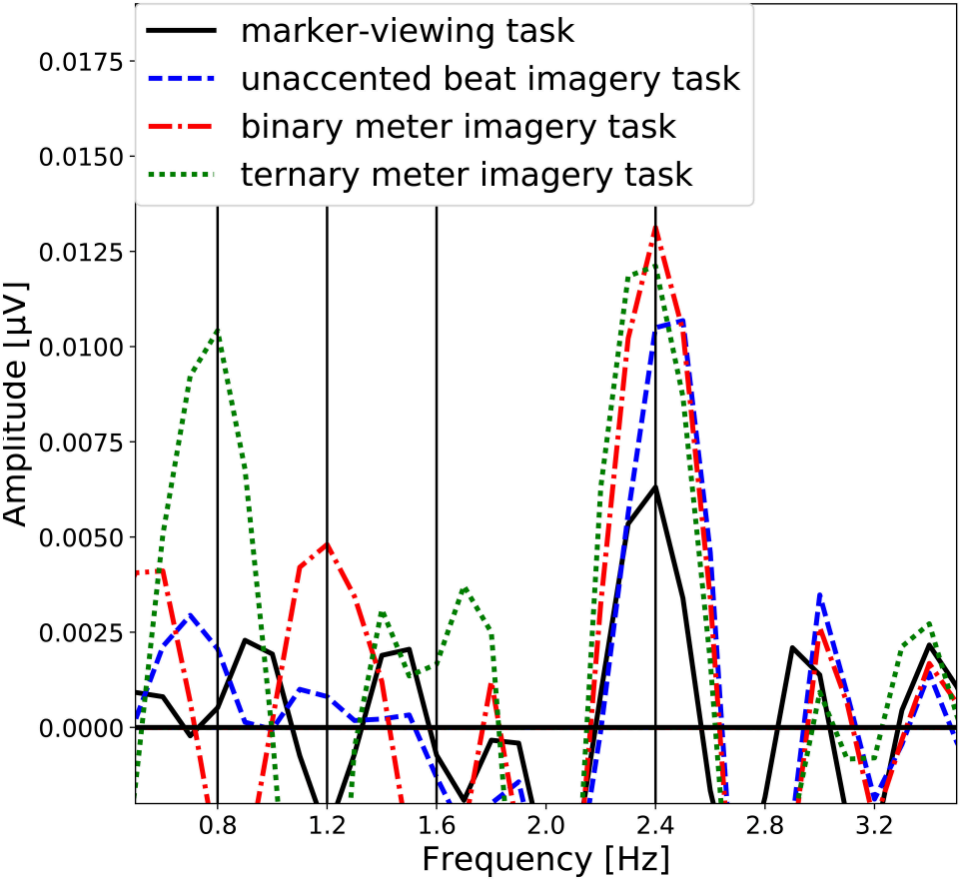
Group-level average of the EEG spectral amplitudes averaged across all scalp electrodes. The EEG spectral amplitudes obtained in the marker-viewing task, the unaccented beat imagery task, the binary meter imagery task, and the ternary meter imagery task are shown in black line, blue dashed line, red dashdot line, and green dotted line, respectively. The vertical lines represent the target frequencies.

The group-level average of the EEG spectral amplitudes are shown in Figure 4. The EEG spectral amplitudes were averaged across all scalp electrodes to enhance the signal component associated with the imagery of beat or meter. In Figure 4, peaks of the EEG amplitude can be observed at the frequency (see Figure 2) corresponding to each task and its harmonic frequencies (unaccented beat imagery: 2.4 Hz; binary meter imagery: 1.2 and 2.4 Hz; ternary meter imagery: 0.8, 1.6, and 2.4 Hz). It is notable that in each imagery task, a peak is exhibited at the frequency of imagined beat and meter.

**Figure 5:**
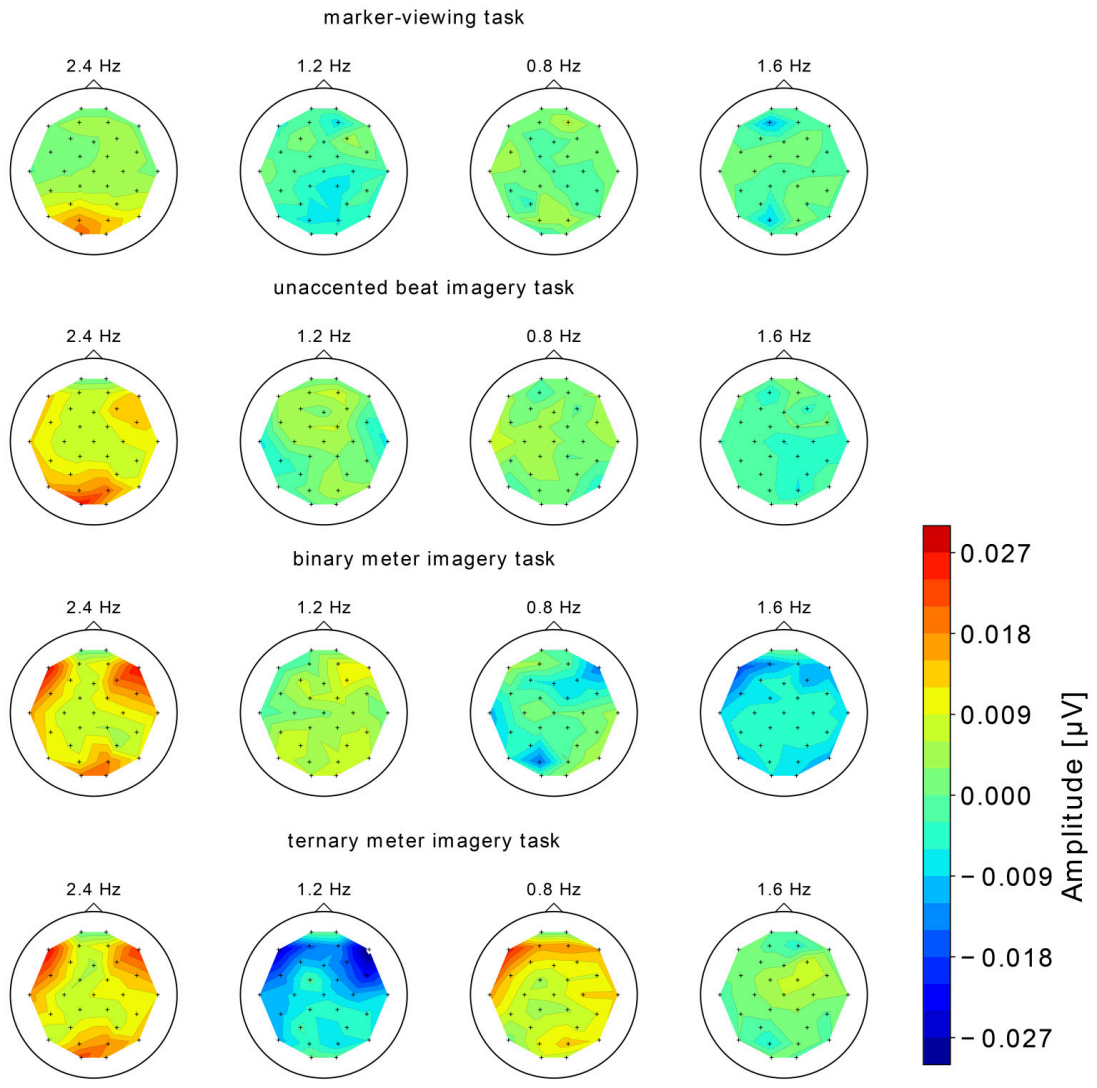
The topographical maps of EEG spectral amplitude at 0.8, 1.2, 1.6, and 2.4 Hz obtained in each of the four tasks. We show the group-level average of the EEG spectral amplitude.

The topographical maps of EEG spectral amplitude at 0.8, 1.2, 1.6, and 2.4 Hz obtained in each of four tasks are shown in Figure 5. In the marker-viewing task, dominant EEG spectral amplitudes at 2.4 Hz were observed in the occipital area. In each of the rhythm imagery tasks, high amplitudes at 2.4 Hz were observed not only in the occipital area but also in the frontal area. In the binary and ternary meter imagery tasks, relatively high amplitudes at 1.2 and 0.8 Hz, respectively, were observed over a widespread area.

### 3.2. Statistical analyses

#### 3.2.1. One-tailed one-sample wilcoxon signed-rank tests

We examined whether EEG spectral amplitudes at the target frequencies were significantly greater than zero. Table 1 shows the results of one-sample Wilcoxon signed-rank tests applied to the EEG spectral amplitudes averaged across all scalp electrodes. Both in the marker-viewing task and in the unaccented beat imagery task, the EEG spectral amplitudes at 2.4 Hz were significantly greater than zero. In the binary meter imagery task, EEG spectral amplitudes at 1.2 and 2.4 Hz were significantly greater than zero. In the ternary meter imagery task, EEG spectral amplitudes at 0.8 and 2.4 Hz were significantly greater than zero.

**Table 1:** Results of one-sample Wilcoxon signed-rank tests that were applied to EEG spectral amplitudes averaged across all scalp electrodes (^*^p < 0.05; ^**^p < 0.01).

**Task**	**Target frequency (Hz)**	***z*-value**	***p*-value**
Marker-viewing	0.8	1.07	0.29
	1.2	0.10	0.92
	1.6	0.37	0.71
	2.4	3.10	0.0020^**^
Unaccented beat imagery	0.8	1.64	0.102
	1.2	0.82	0.41
	1.6	0.27	0.78
	*2.4*	3.10	0.0020^**^
Binary meter imagery	0.8	0.061	0.95
	*1.2*	2.08	0.037^*^
	1.6	0.046	0.96
	2.4	3.10	0.0020^**^
Ternary meter imagery	*0.8*	3.10	0.0020^**^
	1.2	0.012	0.99
	1.6	1.25	1.25
	2.4	2.34	0.020^*^

*The frequency that corresponds with the imagined beat or meter frequency is italicized*.

Next, we inspected the effect of visual timing of 2.4 Hz by examining the amplitudes at 2.4 Hz of the tasks on the occipital and frontal areas. The one-sample Wilcoxon signed-rank tests, which were applied to EEG spectral amplitudes averaged across the four electrodes in the occipital area, showed that the amplitude at 2.4 Hz in each of the rhythm imagery tasks was not significantly different from that in the marker-viewing task (unaccented beat imagery task: *z* = 1.07, *p* = 0.29; binary meter imagery task: *z* = 0.60, *p* = 0.54; ternary meter imagery task: *z* = 0.67, *p* = 0.50). The one-sample Wilcoxon signed-rank tests, which were applied to EEG spectral amplitudes averaged across the 26 non-occipital electrodes, showed that the amplitudes at 2.4 Hz in unaccented beat and binary meter imagery tasks were significantly greater than that in the marker-viewing task (unaccented beat imagery task: *z* = 2.08, *p* = 0.037; binary meter imagery task: *z* = 2.47, *p* = 0.013). The amplitude at 2.4 Hz in the ternary meter imagery task was not significantly different from that in the marker-viewing task (*z* = 1.16, *p* = 0.25).

#### 3.2.2. Friedman test

For each target frequency, a Friedman test was conducted to compare the effect of type of rhythm imagery (unaccented beat, binary meter, and ternary meter imageries) on the EEG spectral amplitudes. The results of the Friedman test for the average EEG amplitudes over all scalp electrodes are as follows. Whereas, the effect of type of rhythm imagery on the EEG spectral amplitudes at 2.4 Hz was not significant, Chi-square = 1.56, *p* = 0.50, the ones at 0.8, 1.2, and 1.6 Hz were significant (0.8 Hz: Chi-square = 12.7, *p* = 0.0018; 1.2 Hz: Chi-square = 6.22, *p* = 0.045; 1.6 Hz: Chi-square = 6.22, *p* = 0.045).

The results of the pairwise Wilcoxon signed-rank tests with respect to tasks are shown in Table 2 and can be summarized as follows. At 0.8 Hz (the frequency of the ternary meter), the amplitude in the ternary meter imagery task was significantly greater than that in the unaccented beat and the binary meter imagery tasks (unaccented beat imagery task: *z* = 2.76, *p* = 0.018; binary meter imagery task: *z* = 3.10, *p* = 0.014). At 1.2 Hz (the frequency of the binary meter), the amplitude in the unaccented beat imagery task and the binary meter imagery tasks was significantly greater than that in the ternary meter imagery task (unaccented beat imagery task: *z* = 2.47, *p* = 0.049; binary meter imagery task: *z* = 2.08, *p* = 0.076). At 1.6 Hz (the frequency of the second harmonic of ternary meter), the amplitude in the ternary meter imagery task was significantly greater than that in the binary meter imagery task (*z* = 2.34, *p* = 0.066).

**Table 2:** The results of the pairwise Wilcoxon signed-rank tests applied to the EEG spectral amplitudes averaged across all scalp electrodes.

**Target frequency (Hz)**	**Compared tasks**	***z*-value**	***p*-value**	**Magnitude relation**
0.8	Unaccented beat imagery vs. Binary meter imagery	0.034	0.97	n.s.
	Unaccented beat imagery vs. Ternary meter imagery	2.76	0.018^*^	Ternary meter imagery > Unaccented beat imagery
	Binary meter imagery vs. Ternary meter imagery	3.10	0.014^*^	Ternary meter imagery > Binary meter imagery
1.2	Unaccented beat imagery vs. Binary meter imagery	0.10	0.92	n.s.
	Unaccented beat imagery vs. Ternary meter imagery	2.47	0.049^*^	Unaccented beat imagery > Ternary meter imagery
	Binary meter imagery vs. Ternary meter imagery	2.08	0.076+	Binary meter imagery > Ternary meter imagery
1.6	Unaccented beat imagery vs. Binary meter imagery	0.047	0.96	n.s.
	Unaccented beat imagery vs. Ternary meter imagery	1.53	0.24	n.s.
	Binary meter imagery vs. Ternary meter imagery	2.34	0.066+	Ternary meter imagery > Binary meter imagery

The results of the pairwise Wilcoxon signed-rank tests applied to the EEG spectral amplitudes averaged across all scalp electrodes (+p < 0.10;

* *p < 0.05)*.

### 3.3. Imagined rhythm classification based on machine learning technique

**Figure 6:**
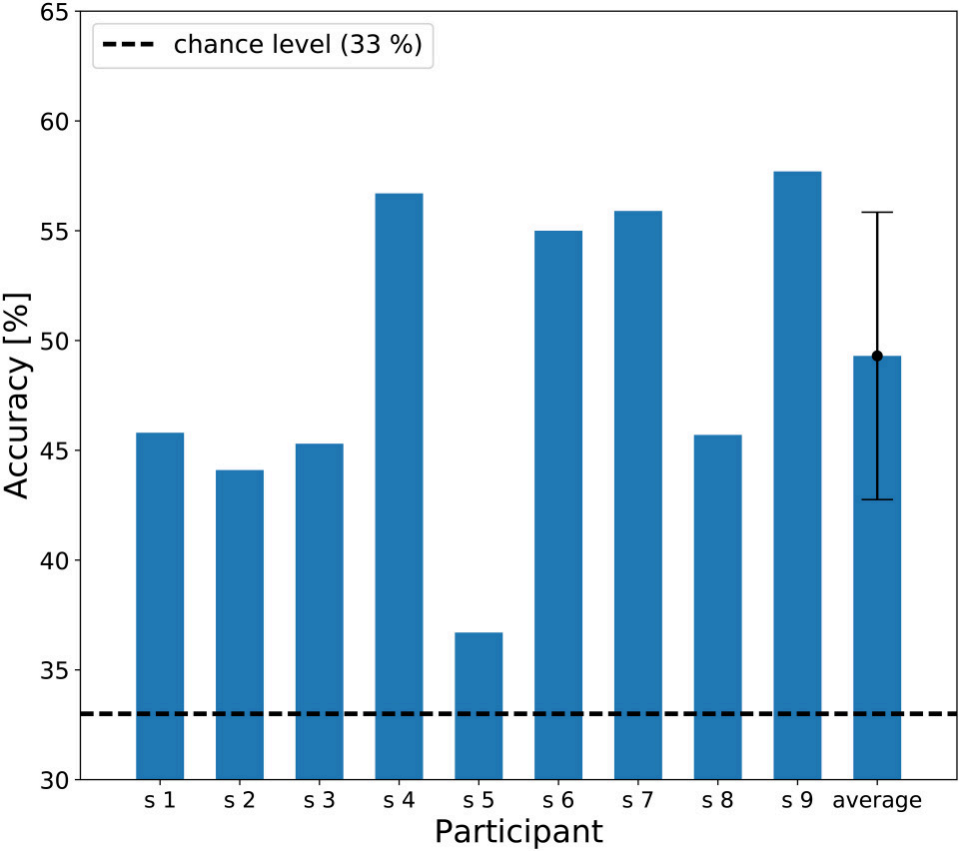
A bar chart of the three-class classification accuracy. The dashed line represents chance level of classification accuracy (33 %). An error bar represents the standard deviation of accuracies.

The individual results of the three-class classification are shown in Figure 6, showing classification accuracies above chance level (33%) for all participants. The best individual result was 57.7% correct, and the averaged accuracy across the participants was 49.3%.

**Figure 7:**
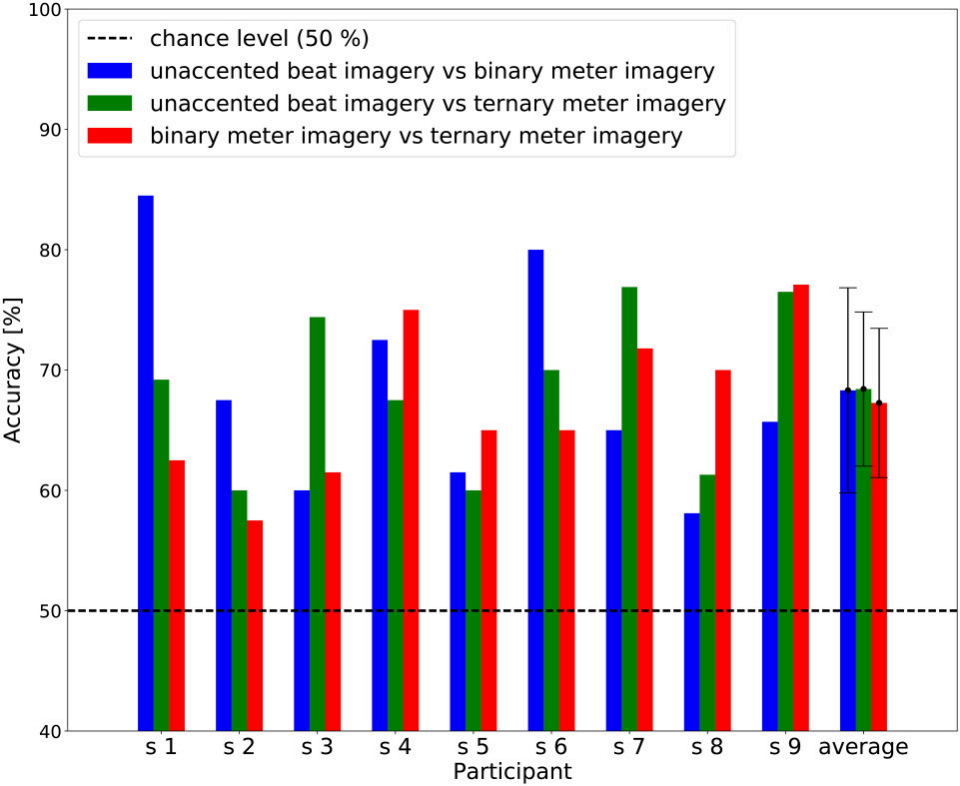
A bar chart of the two-class classification accuracy. The blue, green, and red bars represent individual accuracy of unaccented beat imagery vs. binary meter imagery, unaccented beat imagery vs. ternary meter imagery, and binary meter imagery vs. ternary meter imagery classification, respectively. The dashed line represents chance level of classification accuracy (50 %). Error bars represent the standard deviation of accuracies.

The individual results of the two-class classification are shown in Figure 7, showing classification accuracy above chance level (50%) for all participants. In the unaccented beat imagery vs. binary meter imagery classification, the best individual result was 84.5% correct, and the average accuracy across participants was 68.3%. In the unaccented beat imagery vs. ternary meter imagery classification, the best individual result was 76.9% correct, and the average accuracy across participants was 68.4%. In the binary meter imagery vs. ternary meter imagery classification, the best individual result was 77.1% correct, and the average accuracy across participants was 67.3%.

## 4. Discussion

Our results showed that each rhythm imagery elicited periodic EEG responses oscillating at the beat and meter frequency. These results suggest that mentally imagined beat and meter can be captured by recording EEG signals. Furthermore, the results of classification analysis showed that classification accuracy exceeded chance level in all participants.

### 4.1. Neural oscillation at the beat frequency

We first discuss peaks of the amplitude spectra at 2.4 Hz observed in all the tasks (Figure 4). The one-sample Wilcoxon signed-rank tests indicated the statistical significance of these peaks (Table 1). Moreover, the topographical plots at 2.4 Hz shown in Figure 4 showed that in the marker-viewing task, the EEG amplitudes in the occipital area are stronger than those in the other areas whereas in the rhythm imagery tasks, strong amplitudes were observed not only in the occipital area but also in the frontal area. In other words, at 2.4 Hz in all the tasks, the occipital area showed strong amplitudes. The results showed that in the occipital areas, there is no significant difference in the amplitude between the marker-viewing task and each imagery task. These results suggest that an increase of the EEG amplitude at 2.4 Hz observed in the occipital area may be due to the visual effect of the movie on the visual cortex. In each task, participants kept their eye fixated on the marker, and the black circle came into their sight at a certain interval (2.4 Hz), so it may be suspected that this periodic change of visual information elicited the neural oscillation in the occipital area.

On the other hand, the result of the Wilcoxon signed-rank test for the amplitudes at 2.4 Hz averaged across the 26 non-occipital electrodes showed that there is a significant difference in the amplitude between the unaccented beat or binary meter imagery task and the marker-viewing task. These results suggest that beat imagery by imaging periodic tones elicits neural entrainment to the imagined beat frequency. This is supported by related findings. According to the Dynamic Attending Theory (Jones and Boltz, 1989; Large and Jones, 1999), beat perception is explained as regular fluctuations of attention, and the resonance theory (Large and Kolen, 1994; van Noorden and Moelants, 1999; Large, 2008; Large and Snyder, 2009) hypothesizes that beat perception arises from neural oscillation resonating to the beat frequency. In the current study, participants were asked to periodically imagine the tones synchronized to the moving black circle. This periodic tone imagery may cause attention cycle and beat perception, yielding the observation of neural oscillation at the beat frequency. Another study reported that when participants listened attentively to periodic tones, neural oscillation at the beat frequency was observed in EEG (Nozaradan et al., 2011). However, the neural oscillation at the beat frequency observed in the previous study (Nozaradan et al., 2011) could have contained auditory-evoked potentials. The results of the current study provide stronger evidence for neural entrainment to beat than previous studies (Nozaradan et al., 2011) because neural entrainment to the imagined beat was captured in EEG although participants were not presented with any auditory stimulus.

Another related work investigated brain activity during beat imagery without any auditory stimulus (Jomori et al., 2011) and reported that relative negative potentials were observed in the EEG during beat imagery. Jomori et al. (2011) showed beat imagery appeared as the ERP, while participants imagined a beat during a silent recording period following periodic tones. Negative shift were observed in the period of 100–300 ms after the onset of the imagined beat. However, there was some doubt whether the participants could imagine the beat with an accurate tempo in the absence of a reference tempo during the beat imagery. Moreover, none of the participants had had any experience or special training in musical activities, although Janata and Paroo (2006) found that temporal acuity in auditory imagery was better in participants with more musical training. Our finding on SSEP as responses without auditory stimuli suggested stronger evidence on neural entrainment on imagined beat than the result by Jomori et al. (2011) in the sense that a reference tempo was always given during beat imagery.

As shown in section 3.2.1, the amplitude at 2.4 Hz between the ternary meter imagery task and the marker-viewing task showed no significant difference by the Wilcoxon signed-rank test. Although this result may not be strong support for our hypothesis, this result could be elaborated as follows: It is speculated that the neural oscillation at 2.4 Hz is the sum of the neural entrainment to the imagined beat and the harmonic of the neural entrainment to the imagined meter. In general, the harmonic amplitude of SSEP decreases as the harmonic number increases (Beck et al., 2007). This finding could also be verified with the data measured in this paper. In the ternary meter imagery task, the amplitude at 0.8 Hz (the 1st harmonic) was significantly greater than that at 1.6 Hz (the second harmonic) as a result of the Wilcoxon signed-rank test (*z* = 2.58, *p* = 0.0098), and moreover, the amplitude at 1.6 Hz (the 2nd harmonic) was also significantly greater than that at 3.2 Hz (the 4th harmonic) (*z* = 2.08, *p* = 0.037). These results imply that the amplitude of the 3rd harmonic can be smaller than that of the 2nd harmonic. Thus, at 2.4 Hz, the amplitude of the ternary meter imagery can be smaller than that of the binary meter imagery. This may be supportive of the result of our statistical test. However, in order to examine this issue, it is necessary to analyze EEG during imagination of the meter that has four or more beats.

### 4.2. Neural oscillation at the binary and ternary meter frequencies

In the binary and the ternary meter imagery tasks, the significant peak of the EEG amplitudes at each of the meter frequencies (binary meter; 1.2 Hz, ternary meter; 0.8 Hz) was observed (Figure 4). These results can be explained by the resonance theory (Large and Kolen, 1994; van Noorden and Moelants, 1999; Large, 2008; Large and Snyder, 2009), which predicts the perception of metrical accent as higher-order resonance at the beat frequency. Nozaradan et al. (2011) reported that higher-order resonance at subharmonics of beat frequency was observed during meter imagery. This result is the evidence of the high-order resonance underlying the neural representation of the meter. In the current study, the participants were asked to imagine meter by periodically accenting on the beat. Our results revealed that meter imagery elicited high-order resonance at subharmonics of the beat frequency, and neural entrainment to imagined meter could be captured with EEG although participants were not presented with any auditory stimulus.

The ternary meter imagery task yielded a peak at 1.6 Hz and the second harmonic frequency of 0.8 Hz, as shown in Figure 4. Moreover, the amplitude at 1.6 Hz elicited by the ternary meter imagery was significantly greater than that elicited by the binary meter imagery (Table 2). This can be also confirmed by the classification accuracies for binary vs. ternary imageries (red bar shown in Figure 7) consistently higher than the chance level. A phenomenon similar to this observation was reported in the previous study, as Nozaradan et al. (2011) also observed the significant peak at 1.6 Hz during the ternary meter imagery and discussed two possibilities. One is the effect of harmonics of the ternary meter frequency (i.e., 2 × 0.8 = 1.6 Hz), and the other is the cross-modulation product between beat and ternary meter frequencies (i.e., 2.4 − 0.8 = 1.6 Hz). Differing from Nozaradan et al. (2011), the current experiment reported, the experiment in the present paper never used any auditory stimuli; therefore, our finding supports the hypothesis of the effect of harmonics. Although the ternary meter imagery (0.8 Hz) can affect the amplitude at 1.6 Hz as the second harmonic, the statistical test indicated that there was no significant difference at 1.6 Hz between unaccented beat and ternary meter imageries. This could be explained by the decrease of harmonic amplitudes as discussed in section 4.1.

Next, consider the effects of the unaccented beat imagery appearing at 1.2 Hz lower than the beat frequency (2.4 Hz). According to previous works (Brochard et al., 2003; Bolton, 1894; Martin et al., 2007; Vos, 1973) that investigated the bias of humans in beat perception, this oscillation could be explained by the effect of an involuntary binary meter imagery, which occurred concurrently with the unaccented beat imagery. The related works (Bolton, 1894; Vos, 1973) reported that even though listeners attended isochronic and physically identical tones, they tended to perceive that some tones were accented and that isochronic tones had a metrical structure like binary meter.

### 4.3. Imagined rhythm classification based on machine learning technique

In addition to the conventional statistical tests, the machine learning based classification analysis revealed that the EEG during the auditory imagery of rhythm can be correctly classified at greater than the chance level in all participants (Figures 6, 7). Machine learning techniques were adopted to analyze brain activity in some previous studies (Tucciarelli et al., 2015; Turella et al., 2016). The feature values used for the classification depend on the frequency components contained in the EEG signal; therefore, these results suggest that the frequency components contained in the single trial EEG are different among rhythm imagery tasks. This result with machine learning techniques provides strong support for our findings. Moreover, the CCA-based feature extraction has proven quite effective in detecting steady-state visually evoked potentials, which are responses to periodic visual stimuli (Chen et al., 2015). The result of classification based on CCA suggests that this technique can be applied in novel auditory BMI.

### 4.4. Possible effects of eye movements

Although participants were asked to keep their eyes fixated on the marker and not to move their eyes during the task, the eyes can involuntarily pursue the moving black circles, yielding electrooculogram artifacts that affect the analysis. It is difficult to confirm the existence of eye movement only from EEG; however, we could hypothesize that the entrainment to the meter was not the result from EEG induced from the imagined meter, but the effect of eye movement associated with the imagined rhythm. If this hypothesis was true, the amplitudes closest to the eyes (Fp1 and Fp2) should have been related to a task. We applied the Friedman test to compare the effect of rhythm imagery tasks on the EEG signal amplitudes averaged across Fp1 and Fp2. As a result, the effect of type of rhythm imagery on the EEG spectral amplitudes at 0.8, 1.2, and 1.6 Hz was not significant (0.8 Hz: Chi-square = 3.56, *p* = 0.17; 1.2 Hz: Chi-square = 0.22, *p* = 0.89; 1.6 Hz: Chi-square = 1.56, *p* = 0.46). This rejects the above hypothesis. In other words, there were no eye movements synchronized to the imagined meter. However, to confirm the existence of eye movement, it is necessary to measure the electrooculogram around the eyes.

## 5. Conclusion

In the current study, we investigated neuronal entrainment to beat and meter, which was imagined without any auditory stimulus, and found that auditory imagery of rhythm elicited a periodic EEG response at imagined beat and meter frequency. Our results suggest that imagined beat and meter can be decoded from EEG even in the absence of reference beat sounds. This study represents the first step toward the realization of a method for reconstructing music using brain activity. We should mention a limitation of our experimental design in terms of the behavioral evidence of the imagery, as Zatorre and Halpern (2005) and Hubbard (2010) pointed out the lack of control for the process of imagery in imagery studies. As described in section 2.3, although we checked whether or not participants imagine the meters correctly in a simple manner, the other electrophysiological studies regarding imagined beat (Jomori et al., 2011) or meter (Nozaradan et al., 2011) showed no behavioral evidence of the imagery. Future studies should also consider more appropriate behavioral tasks to make experimental results more reliable. Moreover, we should investigate the relation of imagined rhythm to spontaneous tempo (Large and Gray, 2015).

## Author contributions

HO designed the experiment, conducted the experiment, and wrote the draft of the manuscript. KS initiated the research plan, contributed to the experiment and writing the manuscript. TT also initiated the research plan, approved the experiment, wrote the draft of the manuscript, and approved for the submission as the PI.

### Conflict of interest statement

The authors declare that the research was conducted in the absence of any commercial or financial relationships that could be construed as a potential conflict of interest.

## Supplementary material

Supplementary Video 1 The movie used for the experiment (see Figure 1).

Supplementary Audio 1 The audio used for an instruction of binary meter imagery.

Supplementary Audio 2 The audio used for an instruction of ternary meter imagery.

## References

[B1] Beck D. L. Speidel D. Petrak M. (2007). Auditory steady-state response (ASSR): a beginner's guide. Hear. Rev. 14, 34–37.

[B2] Bolton T. L. (1894). Rhythm. Am. J. Psychol. 6, 145–238. 10.2307/1410948

[B3] Boser B. E. Guyon I. M. Vapnik V. N. (1992). A training algorithm for optimal margin classifiers, in Proceedings of the Fifth Annual Workshop on Computational Learning Theory, COLT'92 (New York, NY: ACM), 144–152. 10.1145/130385.130401

[B4] Brochard R. Abecasis D. Potter D. Ragot R. Drake C. (2003). The “Ticktock" of our internal clock direct brain evidence of subjective accents in isochronous sequences. Psychol. Sci. 14, 362–366. 10.1111/1467-9280.24441 12807411

[B5] Chen X. Wang Y. Nakanishi M. Gao X. Jung T.-P. Gao S. (2015). High-speed spelling with a noninvasive brain–computer interface. Proc. Natl. Acad. Sci. U.S.A. 112, E6058–E6067. 10.1073/pnas.1508080112 26483479 PMC4640776

[B6] Cooper G. Meyer L. (1960). The Rhythmic Structure of Music. Chicago, IL: University of Chicago Press.

[B7] Demiralp T. Ademoglu A. Istefanopulos Y. Gülçür H. (1998). Analysis of event-related potentials (ERP) by damped sinusoids. Biol. Cybernet. 78, 487–493. 10.1007/s004220050452 9711822

[B8] Denby B. Schultz T. Honda K. Hueber T. Gilbert J. M. Brumberg J. S. (2010). Silent speech interfaces. Speech Commun. 52, 270–287. 10.1016/j.specom.2009.08.002

[B9] Donchin E. Spencer K. M. Wijesinghe R. (2000). The mental prosthesis: assessing the speed of a P300-based brain-computer interface. IEEE Trans. Rehabil. Eng. 8, 174–179. 10.1109/86.847808 10896179

[B10] Galambos R. Makeig S. Talmachoff P. J. (1981). A 40-Hz auditory potential recorded from the human scalp. Proc. Natl. Acad. Sci. U.S.A. 78, 2643–2647. 10.1073/pnas.78.4.2643 6941317 PMC319406

[B11] Hotelling H. (1936). Relations between two sets of variates. Biometrika 28, 321–377. 10.1093/biomet/28.3-4.321

[B12] Hubbard T. L. (2010). Auditory imagery: empirical findings. Psychol. Bull. 136, 302–329. 10.1037/a0018436 20192565

[B13] Janata P. Paroo K. (2006). Acuity of auditory images in pitch and time. Percept. Psychophys. 68, 829–844. 10.3758/BF03193705 17076350

[B14] Jomori I. Uemura J. Nakagawa Y. Hoshiyama M. (2011). Event-related potential study of frontal activity during imagination of rhythm. J. Clin. Neurosci. 18, 1687–1689. 10.1016/j.jocn.2011.05.007 22015102

[B15] Jones M. R. Boltz M. (1989). Dynamic attending and responses to time. Psychol. Rev. 96, 459–491. 10.1037/0033-295X.96.3.459 2756068

[B16] Kimura Y. Tanaka T. Higashi H. Morikawa N. (2013). SSVEP-based brain–computer interfaces using FSK-modulated visual stimuli. IEEE Trans. Biomed. Eng. 60, 2831–2838. 10.1109/TBME.2013.2265260 23739780

[B17] Knerr S. Personnaz L. Dreyfus G. (1990). Single-Layer Learning Revisited: A Stepwise Procedure for Building and Training a Neural Network, Berlin; Heidelberg: Springer Berlin Heidelberg.

[B18] Kohavi R. (1995). A study of cross-validation and bootstrap for accuracy estimation and model selection, in Proceedings of the 14th International Joint Conference on Artificial Intelligence - Volume 2, IJCAI'95 (San Francisco, CA: Morgan Kaufmann Publishers Inc.), 1137–1143.

[B19] Kotz S. A. Schwartze M. Schmidt-Kassow M. (2009). Non-motor basal ganglia functions: a review and proposal for a model of sensory predictability in auditory language perception. Cortex 45, 982–990. 10.1016/j.cortex.2009.02.010 19361785

[B20] Large E. W. (2008). Resonating to musical rhythm: theory and experiment, in The Psychology of Time, ed Grondin S. (West Yorkshire: Emerald), 189–232.

[B21] Large E. W. Gray P. M. (2015). Spontaneous tempo and rhythmic entrainment in a bonobo (Pan paniscus). J. Comp. Psychol. 129, 317–318. 10.1037/com0000011 26147705

[B22] Large E. W. Herrera J. A. Velasco M. J. (2015). Neural networks for beat perception in musical rhythm. Front. Syst. Neurosci. 9:159. 10.1037/0033-295X.106.1.119 26635549 PMC4658578

[B23] Large E. W. Jones M. R. (1999). The dynamics of attending: how people track time-varying events. Psychol. Rev. 106, 119–159.

[B24] Large E. W. Kolen J. F. (1994). Resonance and the perception of musical meter. Connect. Sci. 6, 177–208. 10.1080/09540099408915723

[B25] Large E. W. Snyder J. S. (2009). Pulse and meter as neural resonance. Ann. N.Y. Acad. Sci. 1169, 46–57. 10.1111/j.1749-6632.2009.04550.x 19673754

[B26] Lin Z. Zhang C. Wu W. Gao X. (2006). Frequency recognition based on canonical correlation analysis for SSVEP-based BCIs. IEEE Trans. Biomed. Eng. 53, 2610–2614. 10.1109/TBME.2006.886577 17152442

[B27] Martin X. P. Deltenre P. Hoonhorst I. Markessis E. Rossion B. Colin C. (2007). Perceptual biases for rhythm: the mismatch negativity latency indexes the privileged status of binary vs. non-binary interval ratios. Clin. Neurophysiol. 118, 2709–2715. 10.1016/j.clinph.2007.08.019 17950031

[B28] Matsumoto M. Hori J. (2014). Classification of silent speech using support vector machine and relevance vector machine. Appl. Soft. Comput. 20, 95–102. 10.1016/j.asoc.2013.10.023

[B29] Nozaradan S. Peretz I. Missal M. Mouraux A. (2011). Tagging the neuronal entrainment to beat and meter. J. Neurosci. 31, 10234–10240. 10.1523/JNEUROSCI.0411-11.2011 21753000 PMC6623069

[B30] Nozaradan S. Peretz I. Mouraux A. (2012). Selective neuronal entrainment to the beat and meter embedded in a musical rhythm. J. Neurosci. 32, 17572–17581. 10.1523/JNEUROSCI.3203-12.2012 23223281 PMC6621650

[B31] Plourde G. (2006). Auditory evoked potentials. Best Pract. Res. Clin. Anaesthesiol. 20, 129–139. 10.1016/j.bpa.2005.07.012 16634420

[B32] Potter D. D. Fenwick M. Abecasis D. Brochard R. (2009). Perceiving rhythm where none exists: event-related potential (ERP) correlates of subjective accenting. Cortex 45, 103–109. 10.1016/j.cortex.2008.01.004 19027894

[B33] Pritchard W. S. (1981). Psychophysiology of P300. Psychol. Bull. 89, 506–540. 10.1037/0033-2909.89.3.506 7255627

[B34] Repp B. H. (2005). Sensorimotor synchronization: a review of the tapping literature. Psychon. Bull. Rev. 12, 969–992. 10.3758/BF03206433 16615317

[B35] Repp B. H. Su Y.-H. (2013). Sensorimotor synchronization: a review of recent research (2006–2012). Psychon. Bull. Rev. 20, 403–452. 10.3758/s13423-012-0371-2 23397235

[B36] Schaefer R. S. Farquhar J. Blokland Y. Sadakata M. Desain P. (2011). Name that tune: decoding music from the listening brain. Neuroimage 56, 843–849. 10.1016/j.neuroimage.2010.05.084 20541612

[B37] Snyder J. S. Large E. W. (2005). Gamma-band activity reflects the metric structure of rhythmic tone sequences. Cogn. Brain Res. 24, 117–126. 10.1016/j.cogbrainres.2004.12.014 15922164

[B38] Stapells D. R. Linden D. Suffield J. B. Hamel G. Picton T. W. (1984). Human auditory steady state potentials. Ear Hear. 5, 105–113. 10.1097/00003446-198403000-00009 6724170

[B39] Suefusa K. Tanaka T. (2017). A comparison study of visually stimulated brain–computer and eye-tracking interfaces. J. Neural Eng. 14:036009. 10.1088/1741-2552/aa6086 28198356

[B40] Sur S. Sinha V. (2009). Event-related potential: an overview. Indust. Psychiat. J. 18, 70–73. 10.4103/0972-6748.57865 21234168 PMC3016705

[B41] Thaut M. H. Trimarchi P. D. Parsons L. M. (2014). Human brain basis of musical rhythm perception: common and distinct neural substrates for meter, tempo, and pattern. Brain Sci. 4, 428–452. 10.3390/brainsci4020428 24961770 PMC4101486

[B42] Tucciarelli R. Turella L. Oosterhof N. N. Weisz N. Lingnau A. (2015). MEG multivariate analysis reveals early abstract action representations in the lateral occipitotemporal cortex. J. Neurosci. 35, 16034–16045. 10.1523/JNEUROSCI.1422-15.2015 26658857 PMC6605497

[B43] Turella L. Tucciarelli R. Oosterhof N. N. Weisz N. Rumiati R. Lingnau A. (2016). Beta band modulations underlie action representations for movement planning. NeuroImage 136, 197–207. 10.1016/j.neuroimage.2016.05.027 27173760

[B44] van Noorden L. Moelants D. (1999). Resonance in the perception of musical pulse. J. New Music Res. 28, 43–66. 10.1076/jnmr.28.1.43.3122

[B45] Vialatte F.-B. Maurice M. Dauwels J. Cichocki A. (2009). Steady state visual evoked potentials in the delta range (0.5-5 Hz), in Advances in Neuro-Information Processing: 15th International Conference, ICONIP 2008, Auckland, New Zealand, November 25–28, 2008, Part I, eds Köppen M. Kasabov N. Coghill G. (Berlin; Heidelberg: Springer Berlin Heidelberg), 400–407. 10.1007/978-3-642-02490-049

[B46] Vos P. G. (1973). Waarneming van Metrische Toon Reeksen. Nijmegen: Stichting Studentenpers.

[B47] Yamaguchi H. Yamazaki T. Yamamoto K. Ueno S. Yamaguchi A. Ito T. (2015). Decoding silent speech in japanese from single trial EEGS: Preliminary results. J. Comput. Sci. Syst. Biol. 8, 285–291. 10.4172/jcsb.1000202

[B48] Yoshimura N. Nishimoto A. Belkacem A. N. Shin D. Kambara H. Hanakawa T.. (2016). Decoding of covert vowel articulation using electroencephalography cortical currents. Front. Neurosci. 10:175. 10.3389/fnins.2016.00175 27199638 PMC4853397

[B49] Zatorre R. J. Halpern A. R. (2005). Mental concerts: Musical imagery and auditory cortex. Neuron 47, 9–12. 10.1016/j.neuron.2005.06.013 15996544

[B50] Zhu D. Bieger J. Molina G. G. Aarts R. M. (2010). A survey of stimulation methods used in SSVEP-based BCIs. Intell. Neurosci. 2010, 1–12. 10.1155/2010/702357 PMC2833411 20224799

